# Genetic Mechanism of Tissue-Specific Expression of *PPAR* Genes in Turbot (*Scophthalmus maximus*) at Different Temperatures

**DOI:** 10.3390/ijms232012205

**Published:** 2022-10-13

**Authors:** Xinan Wang, Tingting Zhao, Aijun Ma

**Affiliations:** 1 Yellow Sea Fisheries Research Institute, Chinese Academy of Fisheries Sciences, Qingdao 266071, China; 2 Laboratory for Marine Biology and Biotechnology, Pilot National Laboratory for Marine Science and Technology (Qingdao), Qingdao 266071, China; 3 School of Fisheries, Zhejiang Ocean University, Zhoushan 316022, China

**Keywords:** genotype, tissue, interactions, *PPAR* genes, turbot *Scophthalmus maximus*

## Abstract

In this study, we used PCR to measure the levels of the peroxisome proliferator activated receptor genes *PPARα1*, *PPARα2*, *PPARβ,* and *PPARγ* in the intestine, liver, gill, heart, kidney, brain, muscle, spleen, skin, and stomach of turbot (*Scophthalmus maximus*) cultured under different temperature conditions (14, 20, 23, 25, and 28 °C). We used split-split-plot (SSP) analysis of variance, additive main effects and multiplicative interaction (AMMI) analysis, and genotype main effects and genotype × environment interaction (GGE) biplot analysis to evaluate the genotype × tissue interaction effects on gene expression. The results of the SSP analysis of variance showed that temperature and tissue × gene have highly significant (*p* < 0.01) effect on the expression of *S. maximus* *PPAR* genes. The AMMI analysis results revealed that the expression of *PPAR* genes at the appropriate temperature (14 °C) mainly depended on genotype × tissue interaction and tissue effects. Under stress temperatures, genotype effects, tissue effects, and genotype × tissue interaction, all had significant effects on the expression of *PPAR* genes. The contribution of the genotype effect slowly increased with increasing temperature; it increased faster at 20 °C and then slowly declined at 25 °C. The contribution of the tissue effect slowly increased from 14 to 20 °C, where it sharply decreased, and then it stabilized after a slight fluctuation. The contribution of the genotype × tissue interaction effect showed a fluctuating upward trend throughout the experiment, and it had a significant impact on *PPAR* gene expression. The key temperature at which the three effects changed was 20 °C, indicating that it is the limit temperature for active lipid metabolism under high-temperature stress. The GGE biplot analysis results showed that under suitable water temperature, the expression difference of *PPAR* genes in the liver was the largest; at 20 and 23 °C, the expression difference in the gill was the largest; and at 25 and 28 °C, the expression difference in the brain was the largest. Overall, our results suggest that the mechanism responsible for *PPAR* gene expression under the three high temperatures (23, 25, and 28 °C) was relatively consistent, but it differed from that at 20 °C.

## 1. Introduction

The growth and reproduction of fish are closely related to environmental conditions, and temperature is one of the most important environmental factors in the aquaculture environment [1]. This not only affects growth, development, and survival of aquatic animals but also significantly affects their immune system [2,3]. High temperature induces a stress response in fish and causes a series of changes, including changes in genes, mRNA, proteins, and metabolites [4,5,6,7,8], which directly affect the biological functions of cells, tissues, and organs and cause fish growth and metabolism disorders, a decline in disease resistance, and changes in meat quality [1].

Turbot (*Scophthalmus maximus*) is an economically valuable fish species native to the coast of Europe. Since it was introduced into China in 1992, it has become one of the leading varieties of industrially cultured fish along the northern coast of China [9]. Turbot has strict requirements for environmental conditions, especially temperature. Its optimum growth temperature is 14–17 °C, and its maximum lethal temperature is 28–30 °C [1]. Because of its requirement for relatively low temperature, cool underground seawater must be used for breeding during the high-temperature summer months in northern China. However, the limited underground seawater resources cannot meet the needs of breeding, and this increases breeding costs and limits the breeding scope of this important mariculture fish species. Therefore, understanding the heat-resistance mechanism of turbot and cultivating heat-tolerant varieties are very important research goals [1].

Studies have shown that under stress conditions, the stress response of cells is closely related to changes in energy metabolism (glucose and lipid metabolism) [1,10]. High temperature significantly changes the metabolism of lipids and carbohydrates, and the metabolism of lipids plays an important role in resisting high-temperature stress [10]. As one of the three macronutrients required by fish, fat plays an important role in maintaining fish survival and health. Disordered lipid metabolism directly affects the growth, development, and a variety of physiological activities of fish, including stress resistance [11]. Therefore, the study of lipid metabolism has important biological significance. Numerous studies have shown that the lipid metabolic response pathway of fish is precisely regulated [1]. Peroxisome proliferator activated receptors (*PPARs*) are a class of nuclear hormone superfamily receptors. They are one of the main nuclear receptors controlling lipid metabolism, and they play an important role in regulating the transcription of genes related to lipid metabolism [1,12,13,14,15,16].

*PPARs* were first found in the frog *Xenopus laevis* [17] and then were cloned in the mouse *Mus musculus* [18]. Three subtypes of *PPARs* (*PPARα*, *PPARβ* (also known as *PPARδ*), and *PPARγ*) have been identified in mammals, birds, amphibians, and fish [1,12,13,14,15,16]. *PPARα1* and *PPARα2* have been found in fugu (*Takifugu rubripes*), zebrafish (*Danio rerio*), Japanese rice fish (*Oryzias latipes*), turbot, and grass carp (*Ctenopharyngodon idella*) [1,13,14,15,16,17,18,19]. When activated by corresponding ligands in vivo, these receptors form heterodimers with the retinoid X receptor (RXR) and bind to DNA sequences to regulate gene transcription related to glucose and lipid metabolism [1]. Researchers have conducted extensive research on the structure, function, expression mode, and regulation of lipid metabolism of *PPAR* genes in rodents, humans, poultry, and livestock. Genes of *PPARs* are mostly related to lipid transport and metabolism and play an important role in fatty acid oxidation and fat metabolism [18]. *PPARα* and *PPARβ* activate enzymes related to lipolysis and metabolism, regulate the expression of enzymes encoded by genes, and participate in mitochondria *β*-oxidation. *PPARγ* controls fat accumulation and regulates fat and bone formation. Research of the three subtypes of *PPARs* in fish started late. At present, sequence cloning and identification of *PPARs* have been completed in zebrafish [20], *Dicentrarchus labrax* [21], and *T. rubripes* [22]. Because the research of the three subtypes of *PPARs* in fish started late, studies in this field remain at the level of sequence cloning and tissue-specific or development-specific expression pattern analysis.

To explore the heat-resistance mechanism of turbot, Zhao et al. (2020) [1] used fluorescence quantitative PCR (qPCR) to detect the expression of the three subtypes of *PPAR* genes in different tissues of turbot and the expression of *PPAR*s in the kidney of turbot under high-temperature stress. They identified *PPARα*, *PPARβ*, and *PPARγ* in turbot and reported that they may participate in the regulation of lipid metabolism in a tissue-specific way. This study was also the first to report the expression changes of *PPAR* subtypes under temperature stress. However, the same gene often shows different expression levels in different tissues in the same organism. This has been reported in some livestock [23,24,25,26], plants [27], fish [1,28,29,30], and humans [31]. There are also many reports about the tissue-specific expression of *PPAR* genes in various organisms [1,12,13,14,15,32,33,34,35,36,37,38]. The differential expression of the same gene in different tissues can be attributed to a genotype effect, a tissue effect, and/or genotype × tissue interactions. However, the genetic mechanism of tissue-specific expression has not been reported so far.

In this study, we used split-split-plot (SSP) analysis of variance, additive main effects and multiplicative interaction (AMMI) analysis [39], and genotype main effects and genotype × environment interaction (GGE) biplot analysis [40] to evaluate genotype × tissue interaction effects on *PPARα1, PPARα2, PPARβ*, and *PPARγ* genes in turbot cultured under different temperature conditions. The purpose of this study was to identify the genetic mechanism responsible for the differential expression of *PPARs* in different tissues in turbot to apply the formulation of high-temperature-tolerance breeding program.

## 2. Results

**Figure 1:**
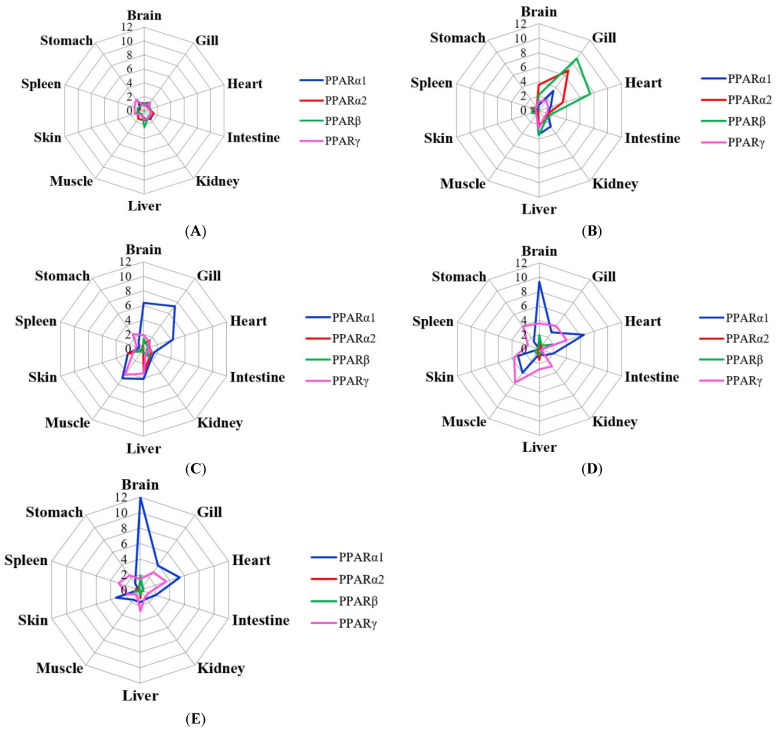
Expression values of PPAR genes in ten tissues at five temperatures ((**A**), 14 °C; (**B**), 20 °C; (**C**), 23 °C; (**D**), 25 °C; (**E**), 28 °C).

Figure 1 shows the expression of *PPARα1*, *PPARα2*, *PPARβ*, and *PPARγ* in different tissues from fish cultured at different temperatures. On the whole, the expression of *PPARα1* and *PPARγ* at 23, 25, and 28 °C were higher than that at the appropriate temperature (14 °C); the expression of PPARα2 and PPARβ peaked at 20 °C.

### 2.1. SSP Analysis of Variance

The results of the SSP analysis of variance are listed in Table 1. Table 1 shows that the *p* values of factors temperature, tissue, gene, temperature × tissue, temperature × gene, tissue × gene, and temperature × tissue × gene were 0.0004, 0, 1 × 10^−7^, 0, 1 × 10^−7^, 1 × 10^−7^, and 1 × 10^−7^, respectively, indicating that the expression of the four *PPAR* genes was significantly (*p* < 0.05) affected by the seven effects (Table 1). 

### 2.2. AMMI Analysis

The AMMI analysis was carried out based on expressions of PPAR genes in different tissues at different temperatures (Supplementary Material Table S1). The results of the AMMI analysis showed that the expression of the four genes was significantly affected by genotype effects, tissue effects, and genotype × tissue interactions at different temperatures (Table 2).

At 14 °C, the results of the AMMI analysis of variance indicated that 0.2545, 27.6400, and 42.7585% of the total sum of squares (SS) were attributable to the genotype, tissue, and genotype × tissue interactions, respectively. IPCA1 and IPCA2 were obtained, which contributed 47.7622 and 35.2611% of the genotype × tissue interaction, respectively.

At 20 °C, the results of the AMMI analysis of variance indicated that 9.8034, 53.4100, and 29.7797% of the total SS were attributable to the genotype, tissue, and genotype × tissue interactions, respectively. IPCA1 and IPCA2 contributed 83.9403 and 11.9052% of the genotype × tissue interaction, respectively.

At 23 °C, the results of the AMMI analysis of variance indicated that 34.3546, 20.1153, and 39.2886% of the total SS were attributable to the genotype, tissue, and genotype × tissue interactions, respectively. IPCA1 and IPCA2 contributed 65.9065 and 26.0857% of the genotype × tissue interaction, respectively.

At 25 °C, the results of the AMMI analysis of variance indicated that 37.28508, 25.4507, and 32.3400% of the total SS were attributable to the genotype, tissue, and genotype × tissue interactions, respectively. IPCA1 and IPCA2 contributed 78.1954 and 20.9852% of the genotype × tissue interaction, respectively.

At 28 °C, the results of the AMMI analysis of variance indicated that 25.8768, 24.9622, and 46.7808% of the total SS were attributable to the genotype, tissue, and genotype × tissue interactions, respectively. IPCA1 and IPCA2 contributed 93.4943 and 6.3767% of the genotype × tissue interaction, respectively.

**Figure 2:**
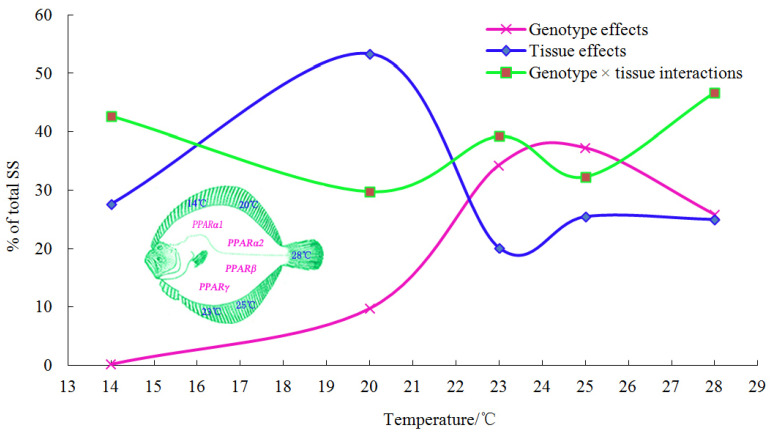
Genotype effect, tissue effect, and genotype × tissue interaction changes with temperature.

The contribution trends of the three effects are shown in Figure 2. The contribution of the genotype effect slowly increased with increasing temperature; it increased faster at 20 °C and then slowly declined at 25 °C. The contribution of the tissue effect slowly increased from 14 °C to 20 °C, where it sharply decreased, and then it stabilized after a slight fluctuation. The contribution of the genotype × tissue interaction effect showed a fluctuating upward trend throughout the experiment, and it had a significant impact on PPAR gene expression.

### 2.3. GGE Biplot Analysis

**Figure 3:**
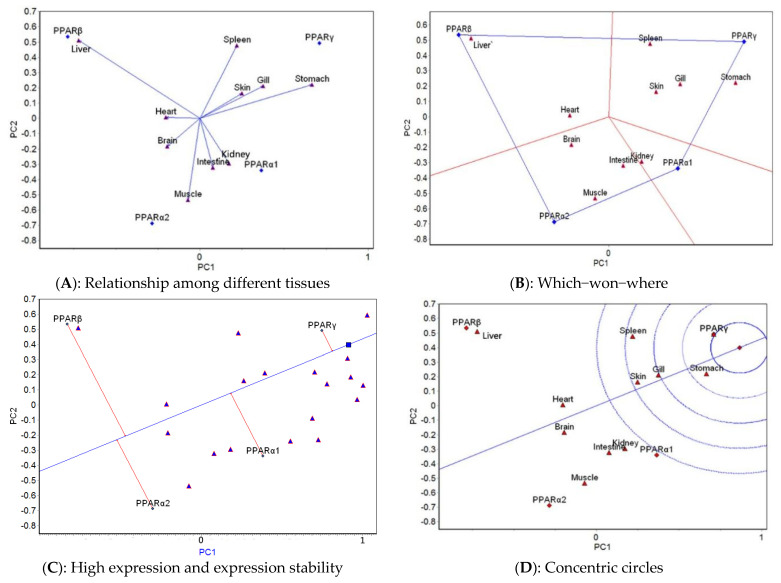
GGE biplots of *PPAR* genes in different tissues at 14 °C.

**Figure 4:**
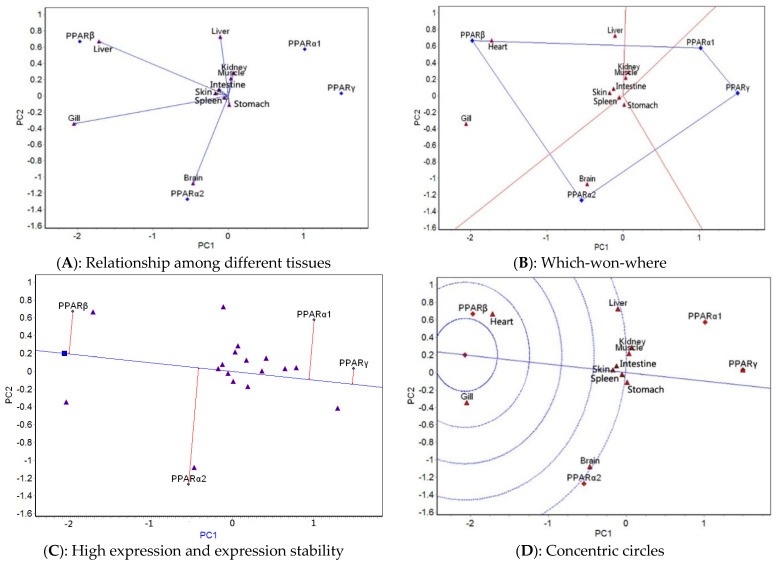
GGE biplots of *PPAR* genes in different tissues at 20 °C.

**Figure 5:**
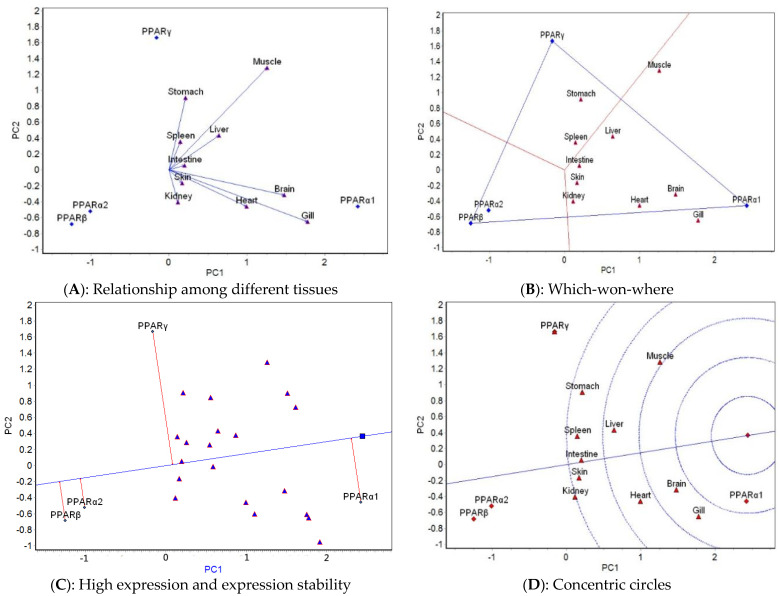
GGE biplots of *PPAR* genes in different tissues at 23 °C.

**Figure 6:**
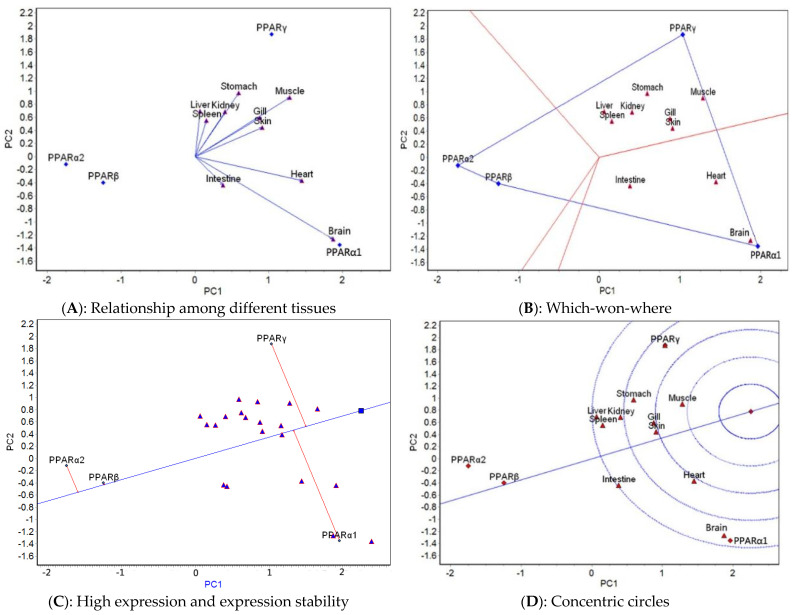
GGE biplots of *PPAR* genes in different tissues at 25 °C.

**Figure 7:**
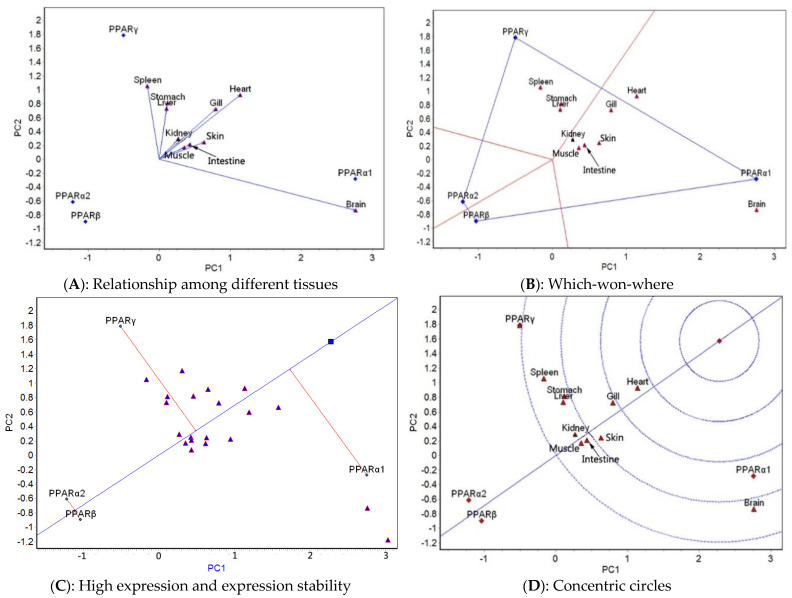
GGE biplots of *PPAR* genes in different tissues at 28 °C.

The GGE biplot analysis was carried out based on the mean expression of four genes in 10 tissues at different temperatures. The relationship among different tissues, which-won-where, high expression and expression stability, and concentric circle GGE biplots for each of the five temperatures tested (Figure 3, Figure 4, Figure 5, Figure 6 and Figure 7) were drawn, respectively, based on the results of the GGE biplot analysis shown in Table 3.

The results of the GGE biplot analysis of the relationship among different tissues (panel A, Figure 3, Figure 4, Figure 5, Figure 6 and Figure 7) mainly reveal the similarity of gene expression among tissues. The included angle of the two line segments indicates the correlation of the gene expression ranking in the tissue represented by the two line segments. When the included angle of the two segments is acute, the gene expression ranking in the two tissues is positively correlated: the smaller the angle, the higher the correlation and the closer the gene expression ranking. When the angle between the two segments is obtuse, the gene expression ranking is negatively correlated in the two tissues, and when the angle between the two segments is a right angle, the gene expression ranking in the two tissues is not related. The length of the line segment indicates the ability of the tissue to distinguish gene expression, with a longer line segment indicating stronger ability to distinguish gene expression. The which-won-where view of the GGE biplot (panel B, Figure 3, Figure 4, Figure 5, Figure 6 and Figure 7) divides the experimental regions according to genotype × tissue interaction and identifies genes with the highest expression level in each region. The gene located at the top corner of the polygon in each region is the gene with the highest expression in this region. The high expression and expression stability biplot (panel C, Figure 3, Figure 4, Figure 5, Figure 6 and Figure 7) shows the genes with high and stable expression. The direction of the transverse oblique line to the ideal value is the approximate average expression of the gene in all tissues: the closer to the ideal value, the higher the average gene expression. The straight line perpendicular to the transverse slash represents the tendency of the gene × tissue interaction, and a greater deviation of the vertical line from the transverse oblique line indicates greater instability of gene expression. The GGE biplot with concentric circles (panel D, Figure 3, Figure 4, Figure 5, Figure 6 and Figure 7) comprehensively evaluates high expression and expression stability based on the distance of various genes from the central point of the genes. A smaller distance indicates higher and more stable gene expression [41].

At 14 °C, the relationship among different tissues biplot (Figure 3A) showed that the angle between skin and gill was the smallest, which indicated that they had basically the same ranking of gene expression. The liver had the longest line segment length, indicating that this tissue had the strongest ability to distinguish the expression of the four genes. The which-won-where view (Figure 3B) divided the 10 tissues into four regions consisting of spleen, skin, gill, and stomach; brain, muscle, and intestine; heart and liver; and kidney. The expression of *PPARγ* was the highest in the spleen–skin–gill–stomach region, that of *PPARα2* was the highest expression in the brain–muscle–intestine region, *PPARβ* had the highest expression in the heart–liver region, and *PPARα1* had the highest expression in the kidney region. The high expression and expression stability biplot (Figure 3C) showed that the expression of *PPARγ* was the highest, followed by *PPARα1*, *PPARβ*, and *PPARα2*; *PPARγ* had the most stable expression, followed by *PPARα1*, *PPARα2*, and *PPARβ*. The concentric circles biplot (Figure 3D) showed that *PPARγ* had the best expression and stability, followed by *PPARα1*, *PPARα2*, and *PPARβ*.

At 20°C, the relationship among different tissues (Figure 4A) showed that the angles between the spleen and gill and between the kidney and muscle were the smallest, which indicated that the ranking of gene expression was the same for each of these pairs. The gill has the longest line segment length, indicating that it had the strongest ability to distinguish the expression of the four genes. The which-won-where view (Figure 4B) divided the 10 tissues into three regions: spleen and muscle; liver, heart, gill, spleen, skin, and intestine; and brain and stomach. The highest expression of *PPARα1* occurred in the spleen–muscle region, that of *PPARβ* was in the liver–heart–gill–spleen–skin–intestine region, and that of *PPARα2* was detected in the brain–stomach region. The high expression and expression stability biplot (Figure 4C) showed that the expression of *PPARβ* was the highest, followed by *PPARα2, PPARα1*, and *PPARγ*; *PPARγ* had the most stable expression, followed by *PPARβ, PPARα1*, and *PPARα2*. The concentric circles biplot (Figure 4D) showed that *PPARβ* had the best expression and stability, followed by *PPARα2*, *PPARα1*, and *PPARγ*.

At 23 °C, the relationship among different tissues (Figure 5A) showed that the angles between the heart and gill and between the spleen and stomach were the smallest, which indicated that the ranking of gene expression was the same for each pair of tissues. The gill had the longest line segment length, indicating that it had the strongest ability to distinguish the expression of the four genes. The which-won-where view (Figure 5B) showed that the 10 tissues were divided into two regions: the spleen and stomach belonged to one area, and the other eight tissues belonged to another area. The expression of *PPARγ* was the highest in the spleen–stomach region, and *PPARα1* expression was the highest in the other area. The high expression and expression stability biplot (Figure 5C) showed that the expression of *PPARα1* was the highest, followed by *PPARγ, PPARα2*, and *PPARβ*; *PPARα2* had the most stable expression, followed by *PPARβ, PPARα1*, and *PPARγ*. The GGE biplot with concentric circles (Figure 5D) showed that *PPARα1* had the best expression and stability, followed by *PPARγ*, *PPARα2*, and *PPARβ*.

At 25 °C, the relationship among different tissues biplot (Figure 6A) showed that the angle between the muscle and gill was the smallest, which indicated that they had basically the same ranking of expression of the four genes. The brain had the longest line segment length, indicating that this tissue had the strongest ability to distinguish the expression of the four genes. The which-won-where view (Figure 6B) showed that the 10 tissues were divided into two regions: intestine, heart, and brain belonged to one area, and the other seven tissues belonged to the other area. *PPARα1* had the highest expression in the intestine–heart–brain region, and *PPARγ* had the highest expression in the other region. The high expression and expression stability view (Figure 6C) showed that the expression of *PPARγ* was the highest, followed by *PPARα1, PPARβ*, and *PPARα2*; *PPARβ* had the most stable expression, followed by *PPARα2, PPARγ*, and *PPARα1*. The GGE biplot with concentric circles (Figure 6D) showed that *PPARγ* had the best expression and stability, followed by *PPARα1*, *PPARβ*, and *PPARα2*.

At 28 °C, the relationship among different tissues biplot (Figure 7A) showed that the angles among the intestine, muscle, and skin as well as those among the kidney, gill, and heart were the smallest, which indicated that the ranking of the expression of the four genes was the same for each set of tissues. The brain had the longest line segment length, indicating that it had the strongest ability to distinguish the expression of the four genes. The which-won-where view (Figure 7B) showed that the 10 tissues were divided into two regions, consisting of spleen, stomach, and liver in one region and the other seven tissues in the second region. The expression of *PPARγ* was the highest in the spleen–stomach–liver region, and that of *PPARα1* was the highest in the other region. The high expression and expression stability biplot (Figure 7C) showed that the expression of *PPARα1* was the highest, followed by *PPARγ, PPARα2*, and *PPARβ*; *PPARβ* had the most stable expression, followed by *PPARα2*. The concentric circles biplot (Figure 7D) showed that *PPARα1* had the best expression and stability, followed by *PPARγ*.

The ranking of high expression, stable expression, and comprehensive evaluation of high and stable expression of *PPAR* genes at different temperatures are shown in Table 4. The top two genes in the comprehensive ranking of the high expression of *PPAR* genes at different temperatures were *PPARγ* and *PPARα1*; for stable expression, they were *PPARα2* and *PPARβ*; and for high and stable expression, they were *PPARγ* and *PPARα1*. Thus, the comprehensive ranking of high and stable expression mainly depended on the high expression of *PPAR* genes.

## 3. Discussion

Many studies have shown that three *PPAR* family members have distinct patterns of tissue distribution [1,12,13,14,15,32,33,34,35,36,37,38]. In mammals, *PPARα* is usually expressed in metabolically active tissues, such as the liver, and it induces the expression of a series of genes related to lipid transport, oxidation, and thermal metabolism. *PPARβ* is widely expressed in the brain, adipose tissue, and skin of the body, but its expression level in the liver is low, and its function is not clear at present. *PPARγ* is present in white adipose tissue and is involved in lipid synthesis [42].

Tissue specificity in the expression of *PPARs* has also been reported in fish [15]. Ibabe et al. (2002) [43] reported that in zebrafish, *PPARα* was mainly expressed in the liver, kidney, intestine, and pancreas; *PPARβ* was expressed in the liver, proximal and distal tubules of the kidney, glomerulus, and pancreas; and *PPARγ* expression in the pancreas, intestine, and gonads was very weak. In the red sea bream *Pagrus major*, three kinds of *PPARs* were widely expressed in the adipose tissue, gills, heart, and hepatocytes of young fish and in the ovaries of adult fish [44]. Studies of brown trout (*Salmo trutta*. *fario*) showed that *PPARα* was mainly present in the white muscle, liver, and heart; *PPARβ* was expressed in the testis, liver, heart, trunk kidney, and white muscle; and *PPARγ* was expressed in the liver and trunk kidney [45]. In their study of sea bream, Leaver et al. (2005) [46] found that *PPARα* was mainly expressed in the heart and liver, *PPARγ* was mainly expressed in the intestine and adipose tissue, and *PPARβ* was expressed in all tissues. In grass carp, *PPARα* was dominant in the liver; *PPARβ* was enriched in the liver, heart, and muscle; and *PPARγ* was rich in the liver but less abundant in the muscle, visceral adipose tissue, and brain [47].

The literature shows that tissue-specific expression of *PPARs* is very common in mammals and fish, and it can be attributed to genotype effects, tissue effects, and genotype × tissue interactions. Therefore, it is of great significance to explore the heat-resistance mechanism of turbot by analyzing the genotype × tissue interactions of *PPAR* genes at different temperatures to elucidate the genetic mechanism responsible for the tissue-specific expression of these genes. In the current study, the tissue was regarded as the “environment”, and the interactions between *PPAR* genes and tissues in turbot were evaluated using SSP, AMMI, and GGE biplot analyses.

The results of the SSP analysis of variance showed that temperature and tissue × gene have highly significant (*p <* 0.01) effect on the expression of *S. maximus PPAR* genes, which indicates that it is of great significance to explore the heat-resistance mechanism of turbot by analyzing the genotype × tissue interactions of *PPAR* genes at different temperatures to elucidate the genetic mechanism responsible for the tissue-specific expression of these genes.

The results of the AMMI analysis revealed that the expression of *PPAR* genes at the appropriate temperature (14 °C) mainly depended on genotype × tissue interactions and tissue effects; however, under stress temperatures, genotype effects, tissue effects, and genotype × tissue interactions, all had significant effects on the expression of *PPAR* genes (Figure 2). Overall, the contribution of genotype effects slowly increased with increasing temperature; it increased faster at 20°C and slowly decreased at 25 °C. The contribution of tissue effects slowly increased and then sharply decreased at 20 °C, and then it reached a stable state after a slight fluctuation. The contribution of genotype × tissue interactions showed a fluctuating upward trend throughout the experiment, and it had a significant impact on *PPAR* gene expression. The trends of the contributions of the three effects to *PPAR* gene expression at the different temperatures clearly showed that 20 °C was the key point at which changes occurred, which may be related to the temperature range in which turbot can adapt. This species has strict requirements for temperature and other environmental indicators. Its suitable growth water temperature is 14–17 °C; its maximum growth temperature is 21–22 °C; it can tolerate temperatures of 25–26 °C but only for short duration; and 28 °C is its lethal temperature [48]. We found that at 20 °C, which is close to the maximum growth temperature of turbot, the mean value of *PPAR* gene expression was the highest (1.9118 U/mg), which indicates that 20 °C is the limit for active lipid metabolism under high-temperature stress. At 14, 20, 23, 25, and 28 °C, the average expression levels of the four *PPAR* genes in the 10 tissues were 1.1166, 1.9118, 1.7727, 1.9112, and 1.5856 U/mg, respectively. The expression levels under high-temperature stress were significantly higher than those under the suitable water temperature. This result was likely related to the regulation of lipid metabolism under high-temperature stress, which involved increasing the overall expression of *PPAR* genes.

The results of the GGE biplot analysis indicated that, on the whole, the ranking of *PPAR* gene expression in different tissues under the appropriate water temperature (14 °C) was more different than that under high-temperature stress, which likely was due to changes of the regulation mechanism of lipid metabolism under high-temperature stress. At 23, 25, and 28 °C, the difference in the *PPAR* gene expression ranking was relatively small. At 14 °C, the expression difference of *PPAR* genes was the greatest in the liver; at 20 and 23 °C, it was the greatest in the gill; and at 25 and 28 °C, it was the greatest in the brain. These results showed that the tissue specificity of *PPAR* gene expression differed at different water temperatures and that under the appropriate water temperature, the liver played a very important role in maintaining normal lipid metabolism. To deal with respiratory function problems at 20 and 23 °C, *PPAR* genes were significantly expressed in the gill. Temperatures of 25 and 28 °C affected the brain, and, therefore, *PPAR* gene expression was high in this tissue.

At different temperatures, the tissue regions with high expression of *PPAR* genes differed, which further indicated that the expression of *PPAR* genes in different tissues at different temperatures has different tissue specificity. The top-ranked genes in terms of high expression at different temperatures were *PPARγ* and *PPARα1*; those for stable expression were *PPARα2* and *PPARβ*; and those for high and stable expression were *PPARγ* and *PPARα1* (Table 4). These were also the patterns for the four high temperatures. The comprehensive ranking of high and stable expression and that of high expression were almost the same, but they differed from that of stable expression. This result indicated that the comprehensive ranking of high and stable expression mainly depended on the high expression of the *PPAR* gene. Overall, the mechanism responsible for *PPAR* gene expression under the three highest temperatures appears to be relatively consistent, but it differs from that at 20 °C. The average value of *PPAR* gene expression was the highest at 20 °C (1.9118 U/mg), which may mean that 20 °C is the temperature limit at which active lipid metabolism can occur under high-temperature stress.

## 4. Materials and Methods

### 4.1. Experimental Materials

The turbot used in the experiment were obtained from Tianyuan Aquatic Limited Corporation (Yantai, China). These fish were artificially bred in a healthy way, with a body mass of 28.19 ± 1.38 g and a body length of 10.1 ± 0.7 cm. Sixty healthy fish with fresh body surface; good vitality; and absence of redness, swelling, or other trauma were selected and put into three experimental barrels with a volume of 2 m^3^, with 20 fish in each barrel. The temperature experiment was carried out after acclimation period for 1 week. During the period of temporary care and during the experiment, the fish were not fed, and the water was changed once a day (the rate of daily water exchange is 50%) and was continuously aerated.

The water-heating process followed that of Ndong et al. (2007) [49] with slight modifications. That is, after 12 h at 14 ± 0.5°C, the test fish were heated to the experimental temperature via a 1 °C increase every 12 h. The hot air pipe was used to heat the seawater in the reservoir tank to a suitable temperature to achieve water temperature regulation. Seawater was pumped into the experimental tank by a water pump. The water flow was 0.05 m^3^/h. We used five experimental temperatures (14, 20, 23, 25, and 28 °C), of which 14 °C was the normal temperature control group [1]. After 12 h at 14 ± 0.5 °C, we removed three fish from each barrel. After being anesthetized with 200 mg/L of MS222 (tricaine methane sulfonate) (Maya Reagent Limited Corporation, Jiaxing, China) [50], we quickly removed the intestine, liver, gill, heart, kidney, brain, muscle, spleen, skin, and stomach and put them on ice and rinsed with 0.9% sodium chloride solution. The remaining turbot were heated to the experimental temperature via a 1 °C increase every 12 h, and the tissues were removed after the fish had been at each experimental temperature for 12 h. Samples were immersed in 10 times the volume of RNA preservation solution (Tiangen Biotech Co., Ltd., Beijing, China) and stored at –80 °C after storage at 4 °C for 24 h. The whole process of the experiment followed the hydrostatic method, and the temperature was controlled by automatic thermostatic heaters to ensure the synchronous temperature increase of each experimental barrel.

### 4.2. Experimental Methods

#### 4.2.1. Total RNA Extraction, Quantification, Integrity Detection, and cDNA Synthesis

The total RNA was extracted from animal tissues (intestine, liver, gill, heart, kidney, brain, muscle, spleen, skin, and stomach) using total RNA Extraction Kits (RNAprep pure Tissue Kit, Tiangen Biotech Co., Ltd., Beijing, China), and the quality and concentration of RNA were detected by 1% agarose gel electrophoresis and Nanodrop 2000 (Thermo Fisher Scientific, Waltham, MA). The RNAs that met the quality criteria (28S:18S = 2:1) were used to synthesize cDNA according to the steps of the reverse transcription kit (TranScript One-Step gDNA Removal and cDNA Synthesis SuperMix, TransGen Biotech Co., Ltd., Beijing, China), and the samples were stored at −20 °C for analysis.

#### 4.2.2. Fluorescence qPCR

We used the housekeeping gene β-actin as the internal reference gene [51] to evaluate the quality of the synthesized cDNA template. We used Primer premier 5.0 software to design fluorescent qPCR primers based on the *PPARα1*, *PPARα2*, *PPARβ*, and *PPARγ* gene sequences of the turbot genome (Assembly GCA_003186165.1). The synthesized primers were subjected to PCR, and the products were sequenced to detect the specificity of primers. Primer synthesis and related sequencing were completed by Sangon Biotech Co., Ltd. (Shanghai, China).

The tissue distribution of *PPAR* RNAs in turbot was detected by fluorescence qPCR. The specific operation method was as follows: The reverse-transcribed cDNAs of the 10 tissues were used as the templates, and the specific primers P1/P2 (*PPARα1*), P3/P4 (*PPARα2*), P5/P6 (*PPARβ*), and P7/P8 (*PPARγ*) of each gene (Table 5) were used on an Applied Biosystems StepOnePlus PCR amplification instrument (Applied Biosystems, Foster City, CA, USA). According to the instructions of the TORO Green qPCR Master Mix (TOROIVD, Shanghai, China), the amplification reaction was carried out using the SYBR Green I chimeric fluorescence method. The PCR system in a 20 μL volume consisted of 10.0 μL of TORO Green qPCR Master Mix, 0.8 μL (10 μMol/L) of each upstream and downstream primer, 2.0 μL of the cDNA template, and 6.8 μL of RNase-free water. The reaction procedure was as follows: pre-denaturation at 95 °C for 60 s followed by 40 cycles of denaturation at 95 °C for 10 s and annealing at 60 °C for 30 s. After the fluorescence qPCR procedure, the relative expression of mRNA was calculated by the △△CT (2^−ΔΔCt^) method [52] based on the Ct values of the *PPARα1*, *PPARα2*, *PPARβ*, *PPARγ*, and *β-actin* genes.

### 4.3. Data Analysis

#### 4.3.1. SSP Analysis of Variance

Referring to Piepho et al. (2018) [53], we used an SSP design for this experiment with temperature as the main-plot factor. We assigned the five experimental temperatures (14, 20, 23, 25, and 28 °C) to the five main plots in each of the three complete replicate blocks. We used tissue as the subplot (or split-plot) factor, with the ten tissues (intestine, liver, gill, heart, kidney, brain, muscle, spleen, skin, and stomach) assigned to ten subplots within each main plot, and *PPAR* genes as the sub-subplot (or SSP) factor, with the four *PPAR* genes (*PPARα1*, *PPARα2*, *PPARβ*, and *PPARγ*) assigned to individual sub-subplots within each subplot. The SSP analysis model is written according to Equation (1):

(1) $$y_{ihjk}=\mu +b_{k}+d_{ihj}+f_{ik}+g_{ihk}+e_{ihjk}$$

where $y_{ihjk}$ is the expression of the *i*-th temperature treatment for the *h*-th tissue and *j*-th *PPAR* gene in the *k*-th complete block; μ, $b_{k}$, and $d_{ihj}$ are the general intercept, effect of the *k*-th block, and *ihj*-th treatment effect, respectively; $f_{ik}$ is the main-plot error associated with the *k*-th block and *i*-th temperature gradient, assumed to be random with zero mean and variance $\sigma_{f}^{2}$; $g_{ihk}$ is the subplot error associated with the *k*-th block, *i*-th temperature, and *h*-th tissue (assumed to be random with zero mean and variance $\sigma_{g}^{2}$); and $e_{ihjk}$ is a residual sub-subplot error with zero mean and variance σ^2^.

#### 4.3.2. AMMI Analysis

The main feature of the AMMI model integrates the analysis of variance and principal component (PC) analysis [39]. The AMMI model for the *g*-th genotype (*PPARα1*, *PPARα2*, *PPARβ*, and *PPARγ*) in the *e*-th tissue (intestine, liver, gill, heart, kidney, brain, muscle, spleen, skin, and stomach) is written according to Equation (2):

(2) $$y_{ge}=\mu +\alpha_{g}+\beta_{e}+\overset{N}{\underset{i=1}{\sum }}\lambda_{n}\gamma_{gn}\delta_{en}+\theta_{ge}\;$$

where $y_{ge}$ is the expression of the four genotypes *g* in tissue *e*; μ, $\alpha_{g}$, and $\beta_{e}$ are the grand mean, average deviation of genotypes (the average value of each genotype minus the grand average value), and average deviation of the tissue (the average of each tissue minus the grand average), respectively; $\lambda_{n}$ is the eigenvalue of the *n-*th interaction PC axis; $\gamma_{gn}$ is the genotype PC score of the *n-*th PC; $\delta_{en}$ is the tissue PC score of the *n*-th PC; *N* is the total number of PC axes; and $\theta_{ge}$ is the residual.

#### 4.3.3. GGE Biplot Analysis

GGE biplot analysis can reveal the complex interactions between different factors [41,54,55]. The gene expression data obtained from different tissue experiments were sorted into a two-way table, in which each value was the average value of the expression of corresponding genes in corresponding tissues (i.e., the phenotype value ($y_{ge}$)). We used singular value decomposition of the first two PCs to fit the GGE biplot model [56], which is written according to Equation (3):

(3) $$y_{ge}=\mu +\beta_{e}+\lambda_{1}\gamma_{g1}\delta_{e1}+\lambda_{2}\gamma_{g2}\delta_{e2}+\theta_{ge}$$

where $y_{ge}$ is the trait mean expression for genotype *g* in tissue *e*; μ, $\beta_{e}$, and $\mu +\beta_{e}$ are the grand mean, main effect of tissue *e*, and mean expression across all genotypes in tissue *e,* respectively; $\lambda_{1}$ and $\lambda_{2}$ are the singular values for the first and second PCs (PC1 and PC2), respectively; $\gamma_{g1}$ and $\gamma_{g2}$ are the eigenvectors of genotype *g* for PC1 and PC2, respectively; $\delta_{e1}$ and $\delta_{e2}$ are the eigenvectors of tissue *e* for PC1 and PC2, respectively; and $\theta_{ge}$ is the residual associated with genotype *g* in tissue *e*.

The SSP, AMMI, and GGE biplot analyses were performed using the DPS Data Processing System (Hangzhou, China) [57].

## 5. Conclusions

In conclusion, at different temperatures, *PPAR* genes in turbot participate in the regulation of lipid metabolism in different tissue-specific ways. Therefore, at different temperatures, the tissues selected to analyze the heat-resistance mechanism of turbot differ in the activities of lipid metabolism-related genes. The gill should be used to study the heat resistance of turbot at 20/23 °C, and the brain should be used at 25/28 °C. Considering that *PPARγ* and *PPARα1* had the best expression and stability, they can be used as indirect selection indexes for temperature tolerance breeding.

## Figures and Tables

**Table 1:** SSP analysis of variance for *S. maximus* high temperature experiment with four *PPAR* genes, five temperature gradients, and ten tissues.

Source of Variation	Sum of Square	Degrees of Freedom	Mean Square	*F*-Value	*p*-Value
Blocks (replicates)	1.2927	2	0.6464		
Temperature	58.603	4	14.651	18.398 **	0.0004
Main-plot error	6.3707	8	0.7963		
Tissue	270.57	9	30.063	128.02 **	0
Temperature × tissue	328.25	36	9.1181	38.83 **	0
Split-plot error	21.134	90	0.2348		
Gene	180.69	3	60.229	249.7 **	1 × 10^−7^
Temperature × gene	321.96	12	26.83	111.23 **	1 × 10^−7^
Tissue × gene	222.42	27	8.2378	34.153 **	1 × 10^−7^
Temperature × tissue × gene	485.05	108	4.4912	18.62 **	1 × 10^−7^
Split-split-plot error	72.361	300	0.2412		

Notes: Asterisks denote that correlations were significant at ** *p* < 0.01.

**Table 2:** AMMI analysis results for *PPARα1*, *PPARα2*, *PPARβ*, and *PPARγ* in different tissues at different temperatures.

Temperatures	Source of Variation	*df*	SS	MS	*F*	Prob.	% of Total SS
	Total	119	21.5253	0.1809			
	Treatment	39	15.2082	0.39	4.9384 **	0	
	Gene	3	0.0548	0.0183	0.2311	0.87444	0.2545
	Tissue	9	5.9496	0.6611	8.3718 **	0	27.6400
14 °C	Interaction	27	9.2039	0.3409	4.317 **	0	42.7585
	IPCA1	11	4.39599	0.39964	5.06101 **	6 × 10^−6^	47.7622
	IPCA2	9	3.2454	0.3606	4.56665 **	7.1 × 10^−5^	35.2611
	Residual	7	1.5625	0.22321			
	Error	80	6.31709	0.07896			
	Total	119	480.995	4.042			
	Treatment	39	447.293	11.4691	27.2247 **	0	
	Gene	3	47.154	15.718	37.3106 **	0	9.8034
	Tissue	9	256.9	28.5444	67.7573 **	0	53.4100
20 °C	Interaction	27	143.239	5.3052	12.5931 **	0	29.7797
	IPCA1	11	120.236	10.9305	25.9463 **	0	83.9403
	IPCA2	9	17.053	1.89477	4.49772 **	8.5 × 10^−5^	11.9052
	Residual	7	5.9508	0.85011			
	Error	80	33.702	0.42127			
	Total	119	370.434	3.1129			
	Treatment	39	347.314	8.9055	30.8149 **	0	
	Gene	3	127.261	42.4204	146.784 **	0	34.3546
	Tissue	9	74.5141	8.2793	28.6483 **	0	20.1153
23 °C	Interaction	27	145.539	5.3903	18.6517 **	0	39.2886
	IPCA1	11	95.9195	8.71996	30.1729 **	0	65.9065
	IPCA2	9	37.9649	4.21832	14.5963 **	0	26.0857
	Residual	7	11.6543	1.6649			
	Error	80	23.12	0.289			
	Total	119	524.157	4.4047			
	Treatment	39	498.347	12.7781	39.6067 **	0	
	Gene	3	195.432	65.1441	201.919 **	0	37.2850
	Tissue	9	133.402	14.8224	45.9432 **	0	25.4507
25 °C	Interaction	27	169.512	6.2782	19.4598 **	0	32.3400
	IPCA1	11	132.551	12.0501	37.3501 **	0	78.1954
	IPCA2	9	35.5725	3.9525	12.2511 **	0	20.9852
	Residual	7	1.38891	0.19842			
	Error	80	25.8101	0.32263			
	Total	119	512.987	4.3108			
	Treatment	39	500.778	12.8405	84.1362 **	0	
	Gene	3	132.745	44.2483	289.934 **	0	25.8768
	Tissue	9	128.053	14.2281	93.229 **	0	24.9622
28 °C	Interaction	27	239.979	8.8881	58.2389 **	0	46.7808
	IPCA1	11	224.367	20.397	133.65 **	0	93.4943
	IPCA2	9	15.3029	1.70032	11.1412 **	0	6.3767
	Residual	7	0.30941	0.0442			
	Error	80	12.2092	0.15261			

Notes: 1 *df*: degree of freedom; SS: sum of squares; MS: mean squares; 2 **: significant at 1% probability level.

**Table 3:** GGE biplot analysis of *PPAR* genes in different tissues at different temperatures.

Temperature	*PPARs* Gene/Tissue	PCA1	PCA2	PCA3	Distance From Center Point (D_i_)
	*PPARα1*	0.3631	-0.3388	0.5799	0.7635
	*PPARα2*	−0.2853	−0.6881	−0.4031	0.8470
	*PPARβ*	−0.7873	0.5357	0.1337	0.9616
	*PPARγ*	0.7094	0.4912	−0.3105	0.9171
	Brain	−0.1959	−0.1842	0.2120	0.3424
	Gill	0.3742	0.2135	0.3533	0.5571
	Heart	−0.2033	0.0081	−0.0187	0.2043
14 °C	Intestine	0.0757	−0.3207	0.0136	0.3298
	Kidney	0.1718	−0.2942	0.1449	0.3703
	Liver	−0.7228	0.5105	−0.049	0.8863
	Muscle	−0.0716	−0.5338	−0.4938	0.7307
	Skin	0.2494	0.1629	−0.0821	0.3090
	Spleen	0.2178	0.4764	−0.3899	0.6530
	Stomach	0.6664	0.2204	−0.1291	0.7137
	*PPARα1*	1.0137	0.5744	0.8333	1.4325
	*PPARα2*	−0.5413	−1.2729	0.2258	1.4016
	*PPARβ*	−1.9714	0.6681	−0.2492	2.0964
	*PPARγ*	1.4990	0.0304	−0.8098	1.7041
	Brain	−0.4701	−1.0778	−0.1075	1.1808
	Gill	−2.0521	−0.3423	0.4680	2.1324
	Heart	−1.7196	0.6663	−0.5255	1.9176
20 °C	Intestine	−0.1222	0.0793	−0.0252	0.1479
	Kidney	0.0731	0.2832	0.8371	0.8867
	Liver	−0.1064	0.7261	0.2317	0.7696
	Muscle	0.0370	0.2154	−0.1444	0.2620
	Skin	−0.1720	0.0282	0.0462	0.1803
	Spleen	−0.0496	−0.0232	0.3393	0.3437
	Stomach	0.0133	−0.1143	−0.2517	0.2767
	*PPARα1*	2.4252	−0.4588	0.0502	2.4687
	*PPARα2*	−1.0131	−0.5212	−1.1106	1.5910
	*PPARβ*	−1.2498	−0.6838	0.9882	1.7339
	*PPARγ*	−0.1622	1.6639	0.0721	1.6734
	Brain	1.4789	−0.3157	0.4705	1.5838
	Gill	1.7790	−0.6510	−0.1629	1.9014
	Heart	0.9966	−0.4567	0.0363	1.0969
23 °C	Intestine	0.1985	0.0565	−0.2898	0.3558
	Kidney	0.1161	−0.4054	−0.0442	0.4240
	Liver	0.6440	0.4309	−1.2183	1.4438
	Muscle	1.2598	1.2849	0.3835	1.8399
	Skin	0.1683	−0.1650	−0.4971	0.5502
	Spleen	0.1445	0.3581	0.0620	0.3911
	Stomach	0.2155	0.9091	−0.0159	0.9345
	*PPARα1*	1.9616	−1.3496	0.1036	2.3833
	*PPARα2*	−1.7486	−0.1206	0.5524	1.8377
	*PPARβ*	−1.2483	−0.4003	−0.6314	1.4550
	*PPARγ*	1.0353	1.8706	−0.0247	2.1382
	Brain	1.8721	−1.2646	0.3754	2.2902
	Gill	0.8757	0.5911	0.0162	1.0567
	Heart	1.4477	−0.3709	−0.5774	1.6021
25 °C	Intestine	0.3812	−0.4361	0.1814	0.6070
	Kidney	0.4074	0.6864	−0.0198	0.7985
	Liver	0.0646	0.6955	0.4082	0.8091
	Muscle	1.2839	0.9041	0.0284	1.5706
	Skin	0.9093	0.4450	−0.0753	1.0152
	Spleen	0.1557	0.5520	−0.1217	0.5864
	Stomach	0.5938	0.9715	0.1387	1.1470
	*PPARα1*	2.7550	−0.2784	0.0241	2.7691
	*PPARα2*	−1.2142	−0.6114	0.3761	1.4105
	*PPARβ*	−1.0348	−0.8981	−0.3546	1.4154
	*PPARγ*	−0.5059	1.7880	−0.045	1.8588
	Brain	2.7607	−0.7314	0.0951	2.8575
	Gill	0.7933	0.7247	−0.1139	1.0805
	Heart	1.1408	0.9294	−0.0646	1.4729
28 °C	Intestine	0.4358	0.2129	0.0948	0.4942
	Kidney	0.2706	0.2908	−0.0118	0.3974
	Liver	0.1054	0.7348	0.4034	0.8449
	Muscle	0.3547	0.1679	−0.1111	0.4079
	Skin	0.6312	0.2448	−0.1734	0.6989
	Spleen	−0.1638	1.0535	0.0795	1.0691
	Stomach	0.1145	0.8142	−0.1518	0.8362

**Table 4:** Ranking of high expression, stable expression, and comprehensive evaluation of high and stable expression of *PPAR* genes at different temperatures.

		Temperatures				
Evaluation Index	Ranking	14 °C	20 °C	23 °C	25 °C	28 °C
High expression	1	*PPARγ*	*PPARβ*	*PPARα1*	*PPARγ*	*PPARα1*
	2	*PPARα1*	*PPARα2*	*PPARγ*	*PPARα1*	*PPARγ*
	3	*PPARβ*	*PPARα1*	*PPARα2*	*PPARβ*	*PPARα2*
	4	*PPARα2*	*PPARγ*	*PPARβ*	*PPARα2*	*PPARβ*
Stable expression	1	*PPARγ*	*PPARγ*	*PPARα2*	*PPARβ*	*PPARβ*
	2	*PPARα1*	*PPARβ*	*PPARβ*	*PPARα2*	*PPARα2*
	3	*PPARα2*	*PPARα1*	*PPARα1*	*PPARγ*	*PPARα1*
	4	*PPARβ*	*PPARα2*	*PPARγ*	*PPARα1*	*PPARγ*
Comprehensive evaluation of high and stable expression	1	*PPARγ*	*PPARβ*	*PPARα1*	*PPARγ*	*PPARα1*
	2	*PPARα1*	*PPARα2*	*PPARγ*	*PPARα1*	*PPARγ*
	3	*PPARα2*	*PPARα1*	*PPARα2*	*PPARβ*	*PPARα2*
	4	*PPARβ*	*PPARγ*	*PPARβ*	*PPARα2*	*PPARβ*

**Table 5:** Primer sequences used in the experiment.

Primer Name	Gene Name	Sequence (5′ to 3′)	Annealing Temperature	Amplification Efficiency
P1(S)	*PPARα*1	CTACTCAAGCCTGGACCTCAACGA	6 °C	93.095%
P2(AS)	*PPARα*1	TCACTGAAGGGACGCCGCA		
P3(S)	*PPARα*2	CCCTGATAACACCTTCCTCTTTCCC	60 °C	93.92%
P4(AS)	*PPARα*2	TGTCTCGGTCGTCTTGATGTCCTG		
P5(S)	*PPARβ*	ACGGCAAAGGCTTCGTTACC	60 °C	96.944%
P6(AS)	*PPARβ*	CTAATGGCAGCAACAAACAGG		
P7(S)	*PPARγ*	ATCTGAAATACTTCCCCCTCACCAC	60 °C	106.12%
P8(AS)	*PPARγ*	GCTGATGCTCGTCATTCCCAA		
P9(S)	*β-actin*	CATGTACGTTGCCATCCAAG	60 °C	97.36%
P10(AS)	*β-actin*	ACCAGAGGCATACAGGGACA		

Notes: S represents the upstream primer and AS represents the downstream primer.

## Data Availability

The data sets analyzed during the current study are available from Supplementary Material.

## Supplementary Materials

The supporting information can be downloaded at: https://www.mdpi.com/article/10.3390/ijms232012205/s1.
