# Supplementary material for: Genetic Mechanism of Tissue-Specific Expression of PPAR Genes in Turbot (Scophthalmus maximus) at Different Temperatures
Source: Int J Mol Sci. 2022 Oct 13;23(20):12205. doi: 10.3390/ijms232012205 (PMC9603064; doi:10.3390/ijms232012205)
Supplement: Supplementary file 1 [file ijms-23-12205-s001.zip › ijms-1896639-Supplementary.pdf]

Table S1. Expressions of *PPAR* target genes in different tissues at different temperatures (Raw data)

Water temperature	Tissue	Gene	Repeat group 1	Repeat group 2	Repeat group 3
14℃	Brain	PPARα1	0.71212861	0.958001316	1.465802507
	Brain	PPARα2	1.303479101	0.728996251	1.052375267
	Brain	PPARβ	0.825332988	1.252263971	0.967553326
	Brain	PPARγ	0.477480895	0.891546723	0.53817281
	Gill	PPARα1	1.435621177	1.550775352	1.26469455
	Gill	PPARα2	0.740149413	0.787301412	0.729915238
	Gill	PPARβ	1.102399708	1.16781413	0.776760593
	Gill	PPARγ	1.590711874	1.104423659	1.532810962
	Heart	PPARα1	0.583515101	0.651790964	0.629296507
	Heart	PPARα2	0.781312458	0.701800539	0.823734247
	Heart	PPARβ	0.534957502	0.832501521	1.245410152
	Heart	PPARγ	0.77745888	0.413048939	0.533035878
	Intestine	PPARα1	1.683875663	1.11836548	1.333160768
	Intestine	PPARα2	1.051077852	1.573352838	1.659369674
	Intestine	PPARβ	1.025668717	1.110856791	0.877677199
	Intestine	PPARγ	0.851074323	1.155400748	1.372372832
	Kidney	PPARα1	1.22834008	1.771985304	1.579890282
	Kidney	PPARα2	1.357547658	1.098187356	1.670762039
	Kidney	PPARβ	1.171682163	0.889176919	0.959846975
	Kidney	PPARγ	1.207099381	1.311615016	1.119905105
	Liver	PPARα1	0.860454244	1.58913024	0.731328885
	Liver	PPARα2	1.28653007	1.297355599	1.613990096
	Liver	PPARβ	2.465731141	2.527143366	2.088608941
	Liver	PPARγ	1.450338374	1.352694981	1.030169913
	Muscle	PPARα1	0.899435365	0.707576349	0.651103319
	Muscle	PPARα2	1.607392245	1.34241235	1.463438588
	Muscle	PPARβ	0.595181399	0.718412588	0.451848738
	Muscle	PPARγ	0.374328087	0.867279544	0.931703117
	Skin	PPARα1	0.574962969	2.067939433	0.841050959
	Skin	PPARα2	0.884482133	0.840175677	1.345677005
	Skin	PPARβ	0.720270961	1.537241177	0.903154573
	Skin	PPARγ	1.508534949	1.709928821	1.150008797
	Spleen	PPARα1	0.246897963	0.956824346	0.817107061
	Spleen	PPARα2	0.645326831	0.680570746	0.922069121
	Spleen	PPARβ	1.035903697	0.802879352	1.202348395
	Spleen	PPARγ	1.2630575	1.902519646	1.309706985
	Stomach	PPARα1	1.610382645	1.347208778	1.216082165
	Stomach	PPARα2	0.868816036	0.962640479	1.195660967
	Stomach	PPARβ	0.573190645	1.289764413	0.761921282
	Stomach	PPARγ	0.42030982	0.849117992	0.490645377
	Brain	PPARα1	0.94618758	0.71462934	0.5630444

	Brain	PPARα2	3.94833892	3.63778487	3.00103674
	Brain	PPARβ	2.74102727	1.40295352	2.336948
	Brain	PPARγ	1.16890094	1.7261946	0.93340864
	Gill	PPARα1	3.28681769	3.13502217	3.5303258
	Gill	PPARα2	6.12261468	8.39216937	6.05627805
	Gill	PPARβ	8.31517225	10.5648564	7.83437549
	Gill	PPARγ	1.50931185	1.49113177	2.2158407
	Heart	PPARα1	1.17970427	2.07751374	1.86591336
	Heart	PPARα2	3.71514582	3.32862572	3.36725571
	Heart	PPARβ	7.14834391	10.4390943	4.83079565
	Heart	PPARγ	1.14272673	1.83813268	1.14215955
20℃	Intestine	PPARα1	1.21213069	1.08182041	1.54869854
	Intestine	PPARα2	1.21776534	1.62120278	1.18001365
	Intestine	PPARβ	1.39844854	1.99478693	1.64845193
	Intestine	PPARγ	1.27410005	1.17659077	1.20884786
	Kidney	PPARα1	2.92641089	2.77182406	2.50095114
	Kidney	PPARα2	1.64515237	1.68880234	1.42835134
	Kidney	PPARβ	1.09656958	1.99213046	1.81609439
	Kidney	PPARγ	1.4564869	1.2847928	0.97534992
	Liver	PPARα1	3.85030873	2.20204097	3.96182514
	Liver	PPARα2	2.19239846	1.56828669	2.30296912
	Liver	PPARβ	2.9174859	4.31495914	3.18625816
	Liver	PPARγ	1.8164438	2.2459202	3.46915914
	Muscle	PPARα1	0.3919254	0.38373189	0.69322946
	Muscle	PPARα2	0.10935675	0.12458956	0.13107187
	Muscle	PPARβ	0.44943791	0.79380402	0.42363947
	Muscle	PPARγ	0.54512653	0.54890958	0.78892163
	Skin	PPARα1	0.6199577	0.56483725	0.59348797
	Skin	PPARα2	1.01914702	0.75122418	0.56970524
	Skin	PPARβ	0.9315431	1.37060635	0.87435117
	Skin	PPARγ	0.26407411	0.56460902	0.42505019
	Spleen	PPARα1	1.16974822	1.0375154	1.15805953
	Spleen	PPARα2	1.27474788	1.13443336	0.69804952
	Spleen	PPARβ	0.95044575	1.04072196	0.71041
	Spleen	PPARγ	0.40257867	0.65082627	0.604616
	Stomach	PPARα1	0.19287472	0.13239446	0.09730096
	Stomach	PPARα2	0.77925646	0.4957681	0.17759936
	Stomach	PPARβ	0.45613358	0.42458534	0.20760804
	Stomach	PPARγ	0.5733757	0.63621403	0.65989248
	Brain	PPARα1	6.233538316	5.66214937	7.21445214
	Brain	PPARα2	0.700110083	0.80966244	0.76508257
	Brain	PPARβ	1.328101446	1.79523471	1.21822611
	Brain	PPARγ	1.808117937	1.97265168	1.86896944
	Gill	PPARα1	7.323633256	7.14526489	7.39196032

	Gill	PPARα2	1.676159045	1.10131456	1.42178821
	Gill	PPARβ	0.693950423	0.96038909	0.57331052
	Gill	PPARγ	1.202810392	1.40981697	1.28140964
	Heart	PPARα1	2.381900296	5.09091696	5.15502645
	Heart	PPARα2	0.806434225	0.58561874	0.91398125
	Heart	PPARβ	0.859314288	0.56044229	0.63020161
	Heart	PPARγ	0.434506429	0.84313214	0.7068811
	Intestine	PPARα1	1.746244973	1.0004416	1.71091249
	Intestine	PPARα2	1.106545866	1.21079912	1.09054715
	Intestine	PPARβ	0.271967766	0.31109309	0.83108781
	Intestine	PPARγ	1.085343583	1.09002498	1.08201116
	Kidney	PPARα1	1.620728995	1.81081708	1.68946838
23℃	Kidney	PPARα2	1.745632707	1.31135642	1.09600651
	Kidney	PPARβ	1.567093906	1.24525967	1.17753852
	Kidney	PPARγ	0.400377694	0.86953251	0.36494036
	Liver	PPARα1	3.820878362	4.78769704	3.68050113
	Liver	PPARα2	4.630208161	2.29126326	2.88594064
	Liver	PPARβ	0.333423288	0.67171481	0.46337394
	Liver	PPARγ	3.54776556	3.2722114	3.12151845
	Muscle	PPARα1	6.01605367	5.52720161	3.29541304
	Muscle	PPARα2	0.098213314	0.0714546	0.09794293
	Muscle	PPARβ	0.374823754	0.42294249	0.36304489
	Muscle	PPARγ	4.217430877	3.92562533	5.12502953
	Skin	PPARα1	1.902620796	2.85138993	2.09358456
	Skin	PPARα2	1.422755086	3.11483904	2.33580669
	Skin	PPARβ	0.949353752	1.23964237	1.51480327
	Skin	PPARγ	1.796005057	1.05669745	1.60407195
	Spleen	PPARα1	0.625975277	0.5123708	0.73722931
	Spleen	PPARα2	0.042310801	0.02024935	0.03940363
	Spleen	PPARβ	0.065725211	0.08019278	0.06923541
	Spleen	PPARγ	0.825742817	1.01286317	1.20047639
	Stomach	PPARα1	0.884022405	1.37197738	1.20825481
	Stomach	PPARα2	0.863004616	0.13382556	0.12936731
	Stomach	PPARβ	0.132980937	0.19581169	0.10067426
	Stomach	PPARγ	2.423761251	3.86720713	1.28931417
	Brain	PPARα1	9.37865208	9.18859045	9.65921255
	Brain	PPARα2	1.47605739	0.81687807	0.93727638
	Brain	PPARβ	1.74674083	2.14796872	1.87354024
	Brain	PPARγ	3.31145695	3.01711667	4.33291999
	Gill	PPARα1	2.71221539	3.1017233	2.75298477
	Gill	PPARα2	0.23615731	0.40708776	0.37797401
	Gill	PPARβ	0.61220088	0.5792258	0.59035241
	Gill	PPARγ	3.61124187	4.53832292	3.68921161
	Heart	PPARα1	5.16133619	6.0114355	8.33872615

	Heart	PPARα2	0.39570173	0.34566671	0.51105771
	Heart	PPARβ	2.5470942	1.56024689	1.6801719
	Heart	PPARγ	4.23842329	3.38541341	4.50379148
	Intestine	PPARα1	1.94221288	2.12058759	2.02294164
	Intestine	PPARα2	0.17457708	0.13906081	0.16430176
	Intestine	PPARβ	0.5523264	0.10479129	0.114626
	Intestine	PPARγ	0.21575975	0.23418629	0.29278338
	Kidney	PPARα1	1.12635255	1.09149577	1.11230618
	Kidney	PPARα2	0.44514359	0.30154453	0.55277012
	Kidney	PPARβ	0.62826617	0.4659169	0.31105573
	Kidney	PPARγ	2.72646941	1.84990947	4.26123061
	Liver	PPARα1	0.54090309	0.75444938	0.75222091
	Liver	PPARα2	1.53583594	1.56754411	1.33882148
25℃	Liver	PPARβ	0.89131932	0.70351259	0.91065059
	Liver	PPARγ	3.17006601	2.27670772	2.98378879
	Muscle	PPARα1	4.16264407	3.58168872	4.3153408
	Muscle	PPARα2	0.38600925	0.44466739	0.30952901
	Muscle	PPARβ	0.94727823	0.7929206	0.4671065
	Muscle	PPARγ	4.14754094	5.44800664	7.6204994
	Skin	PPARα1	2.62469018	2.03413576	4.69483771
	Skin	PPARα2	0.19964203	0.27024017	0.30165235
	Skin	PPARβ	0.31856523	0.7411596	0.97082255
	Skin	PPARγ	4.02762069	2.77310328	4.35475626
	Spleen	PPARα1	0.01776923	0.05435046	0.05752767
	Spleen	PPARα2	0.09413683	0.06113176	0.11291272
	Spleen	PPARβ	0.23013719	0.12938225	0.11149991
	Spleen	PPARγ	1.389697	1.85880302	1.82907563
	Stomach	PPARα1	1.13630377	1.06781276	1.7281955
	Stomach	PPARα2	0.46989446	0.19884899	0.42294046
	Stomach	PPARβ	0.28567354	0.16931391	0.21970815
	Stomach	PPARγ	4.52408388	3.28669941	3.80372511
	Brain	PPARα1	11.3362578	12.7807249	11.7562939
	Brain	PPARα2	1.53398171	1.24984984	1.04683507
	Brain	PPARβ	1.88997093	1.77280725	2.07358182
	Brain	PPARγ	1.30800501	1.65697321	1.34606891
	Gill	PPARα1	4.38650517	4.9897246	2.45353004
	Gill	PPARα2	0.43303149	0.68939121	0.41579507
	Gill	PPARβ	0.67677314	0.56571836	0.34879992
	Gill	PPARγ	2.16919472	3.96464846	2.45114434
	Heart	PPARα1	4.73224036	5.81498359	5.47652253
	Heart	PPARα2	0.61405192	0.34595268	0.48228922
	Heart	PPARβ	0.38394084	0.49150356	0.52268205
	Heart	PPARγ	3.3333666	3.36195256	3.94382856
	Intestine	PPARα1	1.74182875	2.39735351	2.27350761

	Intestine	PPARα2	0.34315115	0.51423369	0.25331426
	Intestine	PPARβ	0.28644021	0.45871074	0.20897243
28℃	Intestine	PPARγ	1.24186286	1.08185736	1.12558422
	Kidney	PPARα1	1.08551131	1.92209468	1.13496567
	Kidney	PPARα2	0.17202467	0.24900794	0.19593927
	Kidney	PPARβ	0.18860003	0.17763008	0.17213883
	Kidney	PPARγ	1.17343834	1.09879126	1.02823523
	Liver	PPARα1	1.1464871	2.5333761	1.05657834
	Liver	PPARα2	1.08168585	1.04005514	1.05067456
	Liver	PPARβ	0.44053267	0.62666263	0.64537747
	Liver	PPARγ	2.83713849	2.98503758	2.35371541
	Muscle	PPARα1	1.5754477	1.28945083	1.77025102
	Muscle	PPARα2	0.04613186	0.03401058	0.04522291
	Muscle	PPARβ	0.14436083	0.14044822	0.13069978
	Muscle	PPARγ	0.63853906	0.80726741	0.78308214
	Skin	PPARα1	0.87350783	0.82182093	0.64343519
	Skin	PPARα2	0.65883762	0.51729554	0.61478981
	Skin	PPARβ	0.99652295	0.71459612	0.58888218
	Skin	PPARγ	1.74998844	1.84575561	1.51863586
	Spleen	PPARα1	0.19738842	0.16760848	0.2032426
	Spleen	PPARα2	0.48668376	0.43203887	0.63211278
	Spleen	PPARβ	0.15405993	0.11829251	0.10957972
	Spleen	PPARγ	3.01626849	3.64881696	2.02097819
	Stomach	PPARα1	1.07457917	1.14310241	0.94508001
	Stomach	PPARα2	0.29988482	0.35912806	0.16592568
	Stomach	PPARβ	0.19267372	0.19210185	0.13410087
	Stomach	PPARγ	2.3472801	2.81529878	1.95961026
